# Can family doctor system improve health service utilization for patients with hypertension and diabetes in China? A difference-in-differences study

**DOI:** 10.1186/s12913-024-10903-6

**Published:** 2024-04-11

**Authors:** Luying Zhang, Peng Zhang, Wen Chen

**Affiliations:** 1 School of Public Health, Fudan University, Shanghai, China; 2 School of Humanities, Shanghai Institute of Technology, 100 Haiquan Road, Fengxian District, Shanghai, China

**Keywords:** Family doctor system, Gatekeepers, Health service utilization, Chronic disease, Difference-in-differences, China

## Abstract

**Background:**

Family doctors, serving as gatekeepers, are the core of primary health care to meet basic health needs, provide accessible care, and improve attainable health. The study objective was to evaluate the impact of the family doctor system on health service utilization among patients with hypertension and diabetes in China.

**Methods:**

Difference-in-Differences (DID) models are constructed to estimate the net effect of the family doctor system, based on the official health management records and medical insurance claim data of patients with hypertension and diabetes in an eastern city of China.

**Results:**

The family doctor system significantly increases follow-up visits (hypertension patients coef. = 0.13, diabetes patients coef. = 0.08, both *p* < 0.001) and outpatient visits (hypertension patients coef. = 0.08, diabetes patients coef. = 0.05, both *p* < 0.001) among the contracted compared to the non-contracted. The proportion of outpatient visits in community health centers among the contracted significantly rose (hypertension patients coef. = 0.02, diabetes patients coef. = 0.04, both *p* < 0.001) due to significantly more outpatient visits in community health centers and fewer in secondary and tertiary hospitals. It also significantly mitigates the increase in inpatient admissions among hypertension patients but not among diabetes patients.

**Conclusions:**

The examined family doctor system strengthens primary care, both by increasing follow-up visits and outpatient visits and promoting a rationalized structure of outpatient utilization in China.

## Background

Primary health care (PHC) addresses meeting the basic health needs of individuals throughout their lives by providing people-centered care in the community, to guarantee the right to the most accessible health care, the utmost equity, and the highest level of attainable health [1]. As the core of primary care, gatekeepers, globally known as family physicians (FP), or general practitioners (GP), and in China family doctors (FD), provide standardized services including preventive and basic medical services to advocate a healthy lifestyle, manage common diseases, and treat patients in primary care setting [2–4].

The effectiveness of gatekeepers on health service utilization has been proven that having gatekeepers promotes more preventive and outpatient service utilization, and decreases unnecessary emergency visits or avoidable inpatient utilization [5–12]. In the United States, the pilot practices found that GP collaborative practice in 27 sites has statistically significantly higher rates of diabetes care, breast cancer screening, and ambulatory primary care visits; lower rates of all-cause hospitalization, emergency department visits, and ambulatory visits to specialists [5]. In Australia, GP care utilization is associated with reduced risk for any emergency department presentations [7]; and using GP services lowers the rate of potentially preventable hospitalizations in people with diabetes [6]. In Portuguese, a study shows that having an assigned GP increases the appropriate use of emergency departments by 1% [8]. In Hungary, GPs motivate participation in cervical cancer screening by 27% of women who initially refused [9]. In Iran, the GP program reduces the number of not only hospitalizations but also specialist visits [10]. In China, Family Physician Integrated Care Program in Taiwan indicates that it might reduce hospital admissions in the long term [11] and a survey of 3148 residents in Hongkong demonstrates people with regular GPs are 2.3% less likely to use emergency services than people without FDs [12].

China has been striving to establish referral systems since major healthcare reform nationwide in 2009, and family doctors serve as gatekeepers to strengthen primary care [13]. Several pilot programs of family doctor system reform were launched in multiple regions, and in 2016, seven departments, led by the State Council’s Medical Reform Office and the National Health Commission, officially launched family doctor system reform nationwide, which provided explicit guidelines on contracted services, content, fees, incentive mechanisms, performance assessment, and technical support [14]. Residents are encouraged to voluntarily contract with family doctors and then contracted residents would be provided with family doctor contract services [15]. The main objectives of the family doctor system were as follows: (1) to expand the family doctor services by enhancing service capacity, improving the quality of basic public health and health management services, ensuring rational drug use, and providing home-based services. (2) to cover patients with chronic diseases as well as vulnerable populations (the elderly, the pregnant, the children, and the disabled), and to provide accessible health management in primary care, delay disease progression, and prevent unnecessary hospitalization for chronic disease patients. (3) to establish a well-functioning referral system with more health service utilization in community health centers rather than in secondary and tertiary hospitals. In 2018, the coverage rate among the key population reached over 71.3% [16], which initially forms the function of gatekeeping in several pilot cities [17].

A few studies in China focused on the impact of family doctors on health service utilization but the net effect was unclear [18–21]. After controlling factors such as age, occupation, income level, and medical insurance type, contracted Chinese residents are proven to utilize more primary medical consultations, as well as chronic disease follow-up services, rehabilitation, and nursing services [19]. A similar result is concluded from research that contracted patients with chronic diseases have higher utilization rates of chronic disease follow-up services than non-contracted patients [18]. Besides that, surveys conducted in Shanghai [20, 22], Hangzhou [23], and Shenzhen [21] found more contracted residents first go to and contact community health centers than non-contracted residents. A higher proportion (51.9%) in the first-visit to primary care was presented among the contracted residents [24]. However, these studies were based on cross-sectional data with a relatively small sample size, and mainly on self-reported services utilization. The net effect of the family doctor system on health service utilization cannot be conducted and more solid data are required. There is also a lack of research to shed light on the change in health service utilization among different institutions, to reflect the role of FD in re-allocating resources and optimizing the referral system.

To evaluate the impact of the family doctor system on health service utilization among patients with hypertension and diabetes, this study presents the change in follow-up service utilization, outpatient service utilization, and inpatient service utilization. The hypotheses of this study were:

1. The family doctor system would promote the follow-up service and the outpatient service utilization as a result reduce or mitigate the increase in the inpatient service utilization among patients with hypertension and diabetes.
2. The family doctor system would increase the health service utilization in community health centers and decrease it in secondary and tertiary hospitals among patients with hypertension and diabetes.

## Methods

### Aim and setting

This study aims to evaluate the impact of the family doctor system on health service utilization among patients with hypertension and diabetes in China.

An eastern city in China was selected as the sample city, since it was one of the earliest pilot cities and implemented the family doctor system on January 1st, 2015. In this city, the coverage of social insurance was 98% in 2015, which was slightly higher than the 95% coverage of social insurance nationwide. According to the family doctor system, the insured residents could voluntarily contract with a family doctor, who cooperates with nurses, and public health practitioners as a team. For the contracted, the uniform service contents of the family doctor system included four aspects: (1) improving the accessibility of timely counseling services in community health centers (2) offering high-quality diagnosis and treatment services, and a green channel for accurate referral services (3) providing integrated healthcare and home-based services (4) implementing health management for chronic patients through regular follow-ups. For the non-contracted, services were provided in a traditional way, where they did not have a designated family doctor during visits and received less comprehensive services such as fewer follow-ups for chronic diseases, home care, and rehabilitation. As a result, the non-contracted may directly seek care at secondary or tertiary healthcare institutions, leading to poor continuity of health services and chronic disease management.

Regarding the family doctor system in this city as an intervention, we chose the years 2014 and 2017 as the pre-treatment period and post-treatment period, considering the effect of this reform. The national family doctor system was designed and formulated based on the practices and experiences of our pilot city, which are fundamentally consistent. Also, we focus on the chronic disease patients who are diagnosed with diabetes and hypertension in our study, because they are the key population of registration in the pilot city and also the mainland China.

### Data sources

We extracted individual-level data in 2014 and 2017 from two databases and then linked the data by pseudonymous patients’ identification. To be specific, the official health management records were provided by the Community Chronic Disease Management System of the city’s Health Information Center, including patients’ individual information such as demographic information and chronic disease follow-up records. Medical insurance claim data during the study period were extracted from the municipal Medical Insurance Bureau, providing information about outpatient and inpatient service utilization, and the level of medical institutions.

**Fig. 1:**
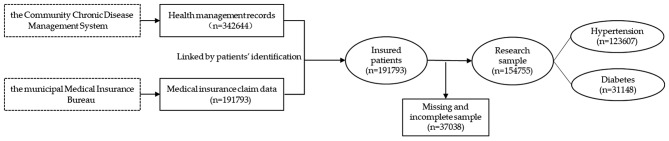
Flow chart of data resource and research sample

This study restricted the research sample according to the following criteria (Fig. 1):

1. Insurers of social medical insurance before January 1, 2014.
2. Participants diagnosed with diabetes and hypertension who registered in community health centers before January 1, 2014.
3. Patients without missing follow-up records in both years.

### Measures

We examined six outcome variables in three sets as follows. (1) We examined the outcome of follow-up service utilization by the average number of annual follow-up visits per capita. The follow-up visits include outpatient visits, phone calls, text messages, and home visits. (2) We examined the four measures of outpatient service utilization: annual outpatient visits per capita, annual outpatient visits in community health centers per capita, annual outpatient visits in secondary and tertiary hospitals per capita, and the proportion of outpatient visits in community health centers. We included the total number of outpatient visits and outpatient visits in different institutions respectively. (3) We examined the outcome of inpatient service utilization, as indicated by annual inpatient admissions per capita.

The key independent variables were two dummy variables *“group*_*i*_*”*, *“time*_*t*_*”*, and their interaction term *“group*_i_*time_*t*_*”*. The dummy variable *“group*_*i*_*”* was created, which equals “1” for the treatment group of contracted patients, who contracted with FDs in 2015 and remained so from then; and “0” for the control group of non-contracted patients, who never contracted with FDs from 2015 to 2017. The other dummy variable *“time*_*t*_*”* was conducted to capture the tendency of change within the group. It equals “0” when the year is 2014 and “1” when the year is 2017.

Other control variables were considered to obtain more robust results. Firstly, unequal health service utilization exists by demographic characteristics [25–27]. To address this issue, age, gender, and insurance type were included. Two main medical insurance in China were the Urban-Rural Resident Basic Medical Insurance (URRBMI), and the Urban Employee Basic Medical Insurance (UEBMI) with further classification of UEBMI for employees and UEBMI for retirees [28]. Besides that, health status and severity of the disease were also controlled, such as comorbidity of diabetes and hypertension, body mass index (BMI), disease duration (in years), as well as respectively average scores of systolic blood pressure (SBP), diastolic blood pressure (DBP) and fasting blood glucose (FBG). The average scores of SBP, DBP, and FBG were calculated by the proportion of normal results to total tests annually and the higher scores the better disease controlled. These are detailed in Table 1.

**Table 1:** Individual characteristics of contracted and noncontracted residents at baseline before and after matching

Subgroups	Hypertension						Diabetes					
	before matching		p value	after matching		p value	before matching		p value	after matching		p value
	contracted	non-contracted		contracted	non-contracted		contracted	non-contracted		contracted	non-contracted	
**N**	103,763	19,844		19,547	19,547		26,934	4214		4121	4121	
**Age**			< 0.001			0.830			< 0.001			0.979
≤ 30	30 (0.0)	31 (0.2)		15 (0.1)	14 (0.1)		20 (0.1)	14 (0.3)		10 (0.2)	12 (0.3)	
30 < age ≤ 40	476 (0.5)	323 (1.6)		296 (1.5)	287 (1.5)		154 (0.6)	107 (2.5)		78 (1.9)	79 (1.9)	
40 < age ≤ 50	4412 (4.3)	1668 (8.4)		1712 (8.8)	1655 (8.5)		1132 (4.2)	420 (10.0)		417 (10.1)	414 (10.0)	
50 < age ≤ 60	22,535 (21.7)	5317 (26.8)		5202 (26.6)	5251 (26.9)		5947 (22.1)	1161 (27.6)		1169 (28.4)	1142 (27.7)	
60 < age ≤ 70	36,420 (35.1)	6423 (32.4)		6450 (33.0)	6349 (32.5)		9868 (36.6)	1303 (30.9)		1278 (31.0)	1276 (31.0)	
70 < age ≤ 80	27,561 (26.6)	4245 (21.4)		4104 (21.0)	4191 (21.4)		7162 (26.6)	869 (20.6)		852 (20.7)	860 (20.9)	
80 < age ≤ 90	11,685 (11.3)	1749 (8.8)		1679 (8.6)	1715 (8.8)		2550 (9.5)	331 (7.9)		306 (7.4)	329 (8.0)	
>90	644 (0.6)	88 (0.4)		89 (0.5)	85 (0.4)		101 (0.4)	9 (0.2)		11 (0.3)	9 (0.2)	
**Gender**			< 0.001			0.332			< 0.001			0.947
Female	57,950 (55.8)	9838 (49.6)		9776 (50.0)	9679 (49.5)		15,071 (56.0)	1921 (45.6)		1893 (45.9)	1889 (45.8)	
Male	45,813 (44.2)	10,006 (50.4)		9771 (50.0)	9868 (50.5)		11,863 (44.0)	2293 (54.4)		2228 (54.1)	2232 (54.2)	
**Comorbidity**			< 0.001			0.905	0.905			< 0.0011	< 0.0011	0.947
No	74,938 (72.2)	16,386 (82.6)		16,138 (82.6)	16,128 (82.5)		4973 (18.5)	1653 (39.2)		1590 (38.6)	1586 (38.5)	
Yes	28,825 (27.8)	3458 (17.4)		3409 (17.4)	3419 (17.5)		21,961 (81.5)	2561 (60.8)		2531 (61.4)	2535 (61.5)	
**Insurance Type**			< 0.001			0.034	0.034		< 0.001			0.946
URRBMI	21,543 (20.8)	396 (2.0)		392 (2.0)	392 (2.0)		4519 (16.8)	58 (1.4)		56 (1.4)	57 (1.4)	
UEBMI for employees	9770 (9.4)	2965 (14.9)		3077 (15.7)	2892 (14.8)		2740 (10.2)	810 (19.2)		800 (19.4)	771 (18.7)	
UEBMI for retirees	72,450 (69.8)	16,483 (83.1)		16,078 (82.3)	16,263 (83.2)		19,675 (73.0)	3346 (79.4)		3265 (79.2)	3293 (79.9)	
**BMI**			< 0.001			0.845			< 0.001			0.477
18.5–24.9	45,536 (43.9)	9895 (49.9)		9702 (49.6)	9737 (49.8)		12,615 (46.8)	2211 (52.5)		2142 (52.0)	2163 (52.5)	
≥ 25	55,141 (53.1)	9374 (47.2)		9291 (47.5)	9245 (47.3)		13,540 (50.3)	1882 (44.7)		1851 (44.9)	1837 (44.6)	
< 18.5	2540 (2.4)	496 (2.5)		469 (2.4)	487 (2.5)		712 (2.6)	114 (2.7)		114 (2.8)	114 (2.8)	
Missing	546 (0.5)	79 (0.4)		85 (0.4)	78 (0.4)		67 (0.2)	7 (0.2)		14 (0.3)	7 (0.2)	
**Disease Duration** ^a^	10.39 (6.85)	10.55 (6.88)	0.004	10.38 (6.97)	10.53 (6.84)	0.380	-	-	-	-	-	-
**Average Scores of SBP** ^a^	0.93 (0.17)	0.94 (0.16)	< 0.001	0.94 (0.16)	0.94 (0.16)	0.342	-	-	-	-	-	-
**Average Scores of DBP** ^a^	0.97 (0.12)	0.97 (0.11)	< 0.001	0.97 (0.11)	0.97 (0.11)	0.530	-	-	-	-	-	-
**Average Scores of FBG** ^b^	-	-	-	-	-	-	0.88 (0.22)	0.90 (0.21)	< 0.001	0.90 (0.20)	0.90 (0.21)	0.909

*Notes* Variables with^a^ are only controlled in the models of hypertension group, while variables with^b^ only in the models of diabetes group. The mean and the (percentage) are shown in the table

### Statistical analysis

A descriptive analysis was conducted and the demographic characteristics at baseline are reported. The t-test was used to compare the difference between contracted and non-contracted patients, with the threshold of statistical significance at *P* < 0.05 (two-tailed).

We assumed that determinants of outcomes remained stable within the same city in two groups over time. Differences-in-differences (DID) approach is adopted to measure the net effect of the family doctor system among patients with hypertension and diabetes using the following model:

$$\eqalign{{Y_{it}}{\mkern 1mu} = {\mkern 1mu} \alpha {\mkern 1mu} & + {\mkern 1mu} {\beta _1}{\mkern 1mu} \cdot \,grou{p_i}{\mkern 1mu} + {\mkern 1mu} {\beta _2}\, \cdot \,tim{e_t}{\mkern 1mu} \cr & + {\mkern 1mu} {\beta _3}\, \cdot \,grou{p_i}{\mkern 1mu} *{\mkern 1mu} tim{e_t}{\mkern 1mu} + {\mkern 1mu} {\beta _{4j}}\, \cdot \,{X_{ij}}{\mkern 1mu} + {\mkern 1mu} {\varepsilon _{it}}\, \cr} $$

Considering that healthcare service utilization follows a positively skewed distribution with a right tail, and the values are discrete and non-negative, we used generalized linear models (GLM) with the Poisson distribution and a log link function to regress Eq. (1) respectively for the hypertension models and the diabetes models. The dummy variable “*group*_*i*_” and “*time*_*t*_” indicated groups and time periods. For six outcomes (*Y*_*i*_), the average treatment effect (ATT) is estimated by comparing the change within the treatment group with the change within the control group during the study period. The parameter of interest is the coefficient of the interaction term *β*_*3*_, which would capture the different changes in Y_it_ among patients in two groups if the family doctor system had an impact. *X*_*ij*_ consists of the control variables as mentioned, and *ε*_*it*_ is the error term [29–31].

As for propensity score matching (PSM), the study included age, gender, comorbidities, insurance type, BMI for all patients, hypertension duration, SBP, and DBP additionally for hypertensive patients, and FBS additionally for diabetes patients as matching variables at the baseline in 2014. Propensity score matching was attempted using 1:1 nearest neighbor matching, 1:3 nearest neighbor matching, kernel matching, and radius matching. It was found that 1:1 nearest neighbor matching yielded the best results but only 31.63% of hypertension patients and 26.46% of diabetes patients were matched. Therefore, the propensity score matching DID (PSM-DID) analysis with 1:1 nearest neighbor matching was chosen for robustness analysis rather than the main analysis.

## Results

### Baseline characteristics

154,755 patients were finally included as the research sample, consisting of 123,607 patients with hypertension and 31,148 patients with diabetes. 130,697 patients contracted with family doctors and 24,058 were non-contracted. As seen in Table 1, contracted patients were older than non-contracted patients, who were more likely to be female and enrolled in the Urban-Rural Resident Basic Medical Insurance. The contracted had higher odds of comorbidity of hypertension and diabetes and tended to be overweight (BMI≥25). For the contracted hypertension patients, the course of hypertension was shorter than the non-contracted with an average of 10.39 years. The average scores and absolute value of SBP and DBP implied that at least 93% of hypertension patients controlled their blood pressure and there was a small but significant difference. As for the diabetes patients, the average score and value of FBG also demonstrated the contracted patients were in worse health status than the non-contracted.

### Difference-in-differences regression

The study presented the annual follow-up visits, outpatient visits (both in community health centers and in secondary and tertiary hospitals), and inpatient admissions, as well as the proportion of outpatient visits in community health centers for the contracted and non-contracted groups in 2014 and 2017, as shown in Table 2. We compared different outcome variables between and within groups and all the differences were significant (*p* < 0.001), and a similar pattern of health service utilization among hypertension and diabetes patients was observed.

**Table 2:** DID results for the impact of the family doctor system on health service utilization in patients with hypertension and diabetes

Outcome	2014		2017		DID	
	Contracted	Non-contracted	Contracted	Non-contracted	coef. (95%CI)	p value
***Hypertension***						
Annual follow-up visits	7.91 (7.88,7.94)	7.02 (6.97,7.07)	8.78 (8.75,8.81)	6.81 (6.75,6.86)	0.13 (0.12,0.13)	< 0.001
Annual outpatient visits	29.13 (29.00,29.26)	21.73 (21.47,21.99)	31.55 (31.41,31.69)	21.97 (21.71,22.24)	0.08 (0.07,0.09)	< 0.001
Annual outpatient visits in community health centers	21.62 (21.53,21.72)	10.90 (10.71,11.08)	25.14 (25.02,25.25)	11.42 (11.22,11.62)	0.09 (0.08,0.11)	< 0.001
Proportion of outpatient visits in community health centers	77.45 (77.31,77.60)	50.11 (49.61,50.60)	82.39 (82.27,82.52)	50.84 (50.34,51.35)	0.02 (0.01,0.03)	< 0.001
Annual outpatient visits in secondary and tertiary hospitals	7.42 (7.36,7.49)	10.70 (10.53,10.87)	6.27 (6.21,6.32)	10.36 (10.19,10.53)	-0.14 (-0.16, -0.13)	< 0.001
Annual inpatient admissions	0.21 (0.21,0.22)	0.24 (0.23,0.25)	0.39 (0.38,0.39)	0.44 (0.42,0.46)	-0.02(-0.04,-0.01)	0.042
***Diabetes***						
Annual follow-up visits	5.70 (5.66,5.74)	5.58 (5.48,5.67)	5.75 (5.72,5.79)	5.13 (5.04,5.23)	0.08 (0.06,0.11)	< 0.001
Annual outpatient visits	33.79 (33.51,34.07)	25.43 (24.81,26.05)	35.93 (35.62,36.23)	25.64 (24.99,26.28)	0.05(0.03,0.08)	< 0.001
Annual outpatient visits in community health centers	24.90 (24.69,25.12)	12.17 (11.72,12.63)	28.45 (28.20,28.70)	12.92 (12.43,13.41)	0.07 (0.04,0.11)	< 0.001
Proportion of outpatient visits in community health centers	76.10 (75.82,76.38)	46.30 (45.22,47.38)	81.61 (81.37,81.85)	47.30 (46.21,48.39)	0.04 (0.02, 0.06)	< 0.001
Annual outpatient visits in secondary and tertiary hospitals	8.78 (8.65,8.91)	13.08 (12.67,13.50)	7.29 (7.17,7.42)	12.43 (12.03,12.84)	-0.13 (-0.16, -0.10)	< 0.001
Annual inpatient admissions	0.28 (0.27,0.28)	0.35 (0.32,0.38)	0.48 (0.46,0.49)	0.57 (0.53,0.62)	-0.09(-0.10, 0.01)	0.124

*Notes* Eq. (1) was estimated by the GLM models with all the other variables mentioned before being controlled. It should be noticed that disease duration, average scores of SBP and DBP were only controlled in the models of the hypertension group, and average score of FBG only in the model of the diabetes group. The coefficient and the (95% CI) are shown in the table

Compared with the non-contracted patients, the contracted significantly utilized more follow-up services and outpatient services both in 2014 and 2017, with a higher proportion of outpatient visits to community health centers, and the difference between groups became greater in 2017. Fewer outpatient visits in secondary and tertiary hospitals and inpatient admissions remained among the contracted from 2014 to 2017.

From 2014 to 2017, for contracted patients, there was a tendency that follow-up visits, outpatient visits, and outpatient visits in community health centers and its proportion, and inpatient admissions steadily increased while outpatient visits in secondary and tertiary hospitals decreased. The contracted group exhibited a greater increase in outpatient visits in community health centers compared to the overall increase in outpatient visits. During the same period, a slight but significant increase in outpatient visits, outpatient visits in community health centers, and its proportion were observed among non-contracted hypertension and diabetes patients, while follow-up visits and outpatient visits in secondary and tertiary hospitals among them declined. Furthermore, the non-contracted group experienced an increase in inpatient admissions from 2014 to 2017, and the magnitude of this increase was greater than that observed in the contracted group.

We identified a statistically significant impact of the family doctor system on health service utilization in patients with both diseases with the main DID results shown in Table 2. After controlling other factors, the family doctor system increased the follow-up service and outpatient service utilization. The results of DID models indicate that the contracted patients increased the number of annual follow-up visits (hypertension patients coef. = 0.13, diabetes patients coef. = 0.08, both *p* < 0.001), and outpatient visits (hypertension patients coef. = 0.08, diabetes patients coef. = 0.05, both *p* < 0.001).

In addition, the structure of outpatient service utilization has changed. More contracted patients went to community health centers for outpatient visits (hypertension patients coef. = 0.09, diabetes patients coef. = 0.07, both *p* < 0.001). The proportion of outpatient visits in community health centers among hypertension and diabetes patients also significantly rose (hypertension patients coef. = 0.02, diabetes patients coef. = 0.04, both *p* < 0.001). At the same time, outpatient visits in secondary and tertiary hospitals decreased with a greater magnitude in both disease groups (hypertension patients coef. = -0.14, diabetes patients coef. = -0.13, both *p* < 0.001).

As for inpatient admissions, the family doctor system may have a lower increase in annual inpatient admissions for contracted patients, but the results are mixed. The results of DID models demonstrate that among hypertensive patients, the family doctor system had a lower increase in annual inpatient admissions of contracted patients compared to non-contracted patients (coef. = -0.02, *p* = 0.042). Among diabetes patients, the contracted patients also had less annual inpatient admissions compared to non-contracted patients, but the lower increase is not statistically significant (coef. = -0.09, *p* = 0.124).

### Robustness analysis

**Fig. 2:**
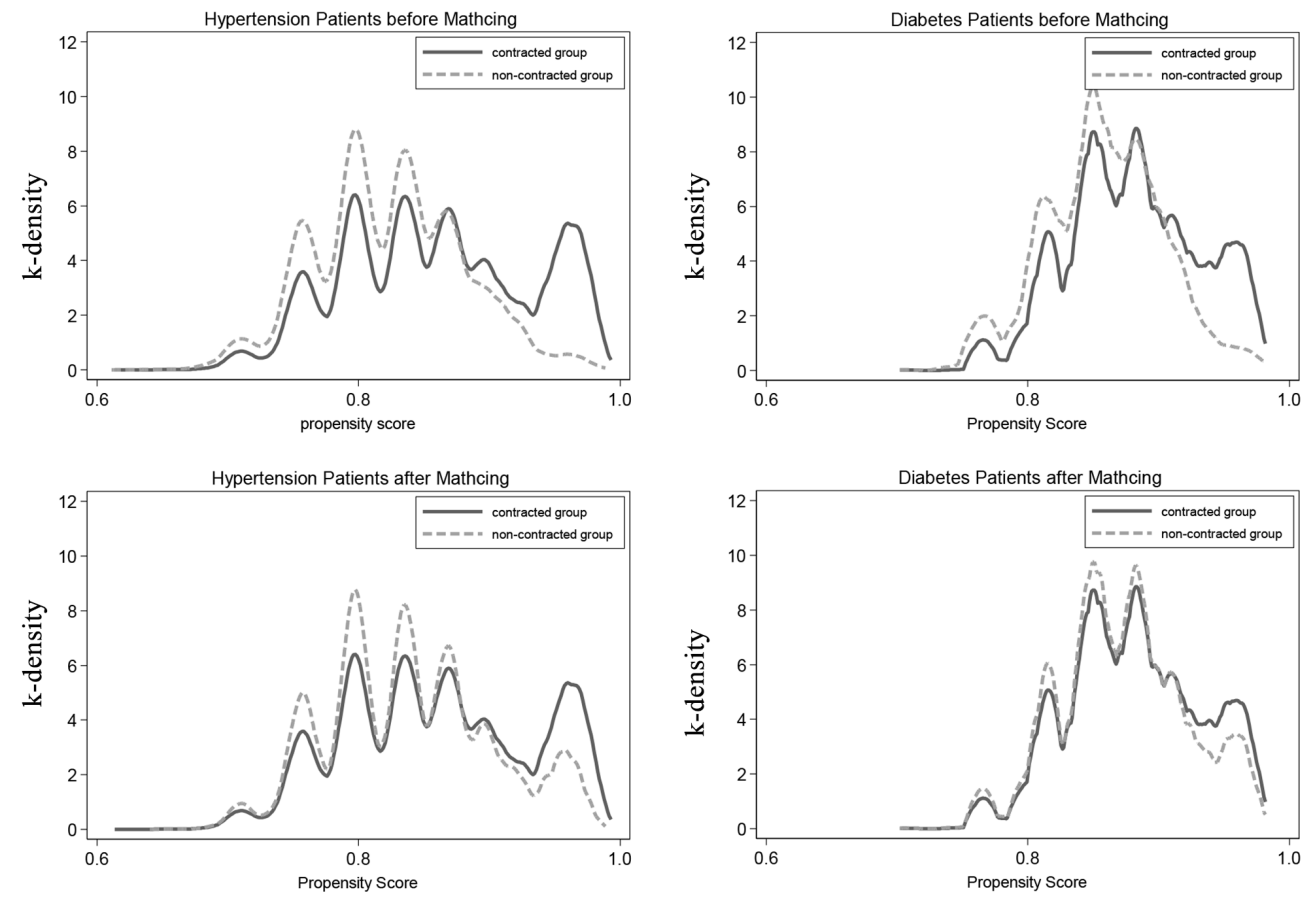
The k-density plots before and after matching for hypertension and diabetes patients

PSM-DID was conducted as the robustness test due to a large number of unmatched samples. After comparing the matching results of four different methods by the reduced bias and t-test of each variable before and after matching, 1:1 nearest matching with logit regression was selected as the best method and the k-density plots before and after matching are shown in Fig. 2. After 1:1 nearest matching, a total of 47,336 patients (39,094 hypertension patients and 8242 diabetes patients) were propensity score matched and two groups were balanced (Table 1).

The description of outcome variables for the matched contracted and non-contracted groups in 2014 and 2017 and the full results of PSM-DID are presented in Table 3. Consistent with the main analysis results, the differences observed in the comparison of outcome variables between and within the matched groups remained statistically significant (*p* < 0.001). The magnitude of differences between and within groups was similar to that observed in the main analysis. The PSM-DID results showed slightly small estimates for some outcome measures (follow-up visits among hypertension and diabetes patients, and the proportion of outpatient visits in community health centers among diabetes patients). However, all the estimates for the outcome measures were still significant, so the main DID results withstood the robustness test.

**Table 3:** PSM-DID results for the impact of the family doctor system on health service utilization in patients with hypertension and diabetes

Outcome	2014		2017		DID	
	Contracted	Non-contracted	Contracted	Non-contracted	coef. (95%CI)	p value
***Hypertension***						
Annual follow-up visits	7.16 (7.10,7.22)	7.03 (6.98,7.08)	7.88 (7.82,7.94)	6.83 (6.77,6.89)	0.12 (0.12,0.13)	< 0.001
Annual outpatient visits	29.74 (29.44,30.04)	21.73 (21.47,21.99)	31.65 (31.32,31.97)	22.00 (21.73,22.26)	0.08 (0.07,0.09)	< 0.001
Annual outpatient visits in community health centers	21.48 (21.25,21.70)	10.91 (10.72,11.09)	24.36 (24.10,24.62)	11.44 (11.24,11.64)	0.09 (0.08,0.11)	< 0.001
Proportion of outpatient visits in community health centers	75.11 (74.78,75.45)	50.21 (49.71,50.71)	79.54 (79.24,79.84)	50.91 (50.40,51.42)	0.02 (0.01,0.03)	< 0.001
Annual outpatient visits in secondary and tertiary hospitals	8.17 (8.02,8.32)	10.69 (10.52,10.86)	7.12 (6.98,7.26)	10.36 (10.19,10.53)	-0.14 (-0.16, -0.13)	< 0.001
Annual inpatient admissions	0.21 (0.20,0.22)	0.24 (0.23,0.25)	0.36 (0.34,0.37)	0.44 (0.42,0.46)	-0.02 (-0.05, -0.01)	0.013
***Diabetes***						
Annual follow-up visits	5.56 (5.47,5.66)	5.58 (5.49,5.67)	5.66 (5.57,5.75)	5.12 (5.03,5.22)	0.07 (0.06,0.106)	< 0.001
Annual outpatient visits	33.45 (32.74,34.17)	25.34 (24.72,25.95)	35.37 (34.63,36.12)	25.72 (25.07,26.37)	0.05 (0.03,0.08)	< 0.001
Annual outpatient visits in community health centers	23.85 (23.30,24.39)	12.22 (11.77,12.66)	26.98 (26.39,27.57)	13.00 (12.50,13.49)	0.07 (0.04,0.10)	< 0.001
Proportion of outpatient visits in community health centers	73.11 (72.39,73.83)	46.76 (45.69,47.82)	78.66 (78.02,79.29)	47.38 (46.27,48.48)	0.03 (0.02,0.06)	< 0.001
Annual outpatient visits in secondary and tertiary hospitals	9.49 (9.15,9.83)	12.95 (12.54,13.36)	8.20 (7.88,8.51)	12.45 (12.04,12.86)	-0.13 (-0.16, -0.10)	< 0.001
Annual inpatient admissions	0.27 (0.24,0.29)	0.38 (0.35,0.41)	0.44 (0.40,0.47)	0.58 (0.53,0.62)	-0.09 (-0.10, -0.03)	0.128

*Notes* The study included age, gender, comorbidities, insurance type, BMI for all patients, hypertension disease duration, SBP, and DBP additionally for hypertensive patients, and FBS additionally for diabetes patients as matching variables for 1:1 nearest matching at the baseline in 2014. Equation (1) was estimated by the GLM models with all the other variables mentioned before being controlled. It should be noticed that disease duration, average scores of SBP and DBP were only controlled in the models of the hypertension group, and average score of FBG only in the model of the diabetes group. The coefficient and the (95% CI) are shown in the table

## Discussion

### Main findings

The study adds to the evidence of the impact of FD on health service utilization, which reveals that the family doctor system increases follow-up visits among contracted patients as well as outpatient visits, while it mitigates the increase in inpatient admissions. It also increases the health service utilization in community health centers and decreases it in secondary and tertiary hospitals among patients with hypertension and diabetes, implying a better structure of outpatient utilization. This paper not only extends previous study findings on the impact of the family doctor system but also enriches practical evidence for developing countries with similar contexts.

The results show that the family doctor system increased the follow-up service and outpatient service utilization and mitigated the increase in inpatient admissions, suggesting that contracted patients may shift their utilization from the inpatient care to the outpatient setting. Consistent with other studies, our study found that the family doctor system increases follow-up visits and outpatient visits among contracted patients. Compared to residents without FDs, contracted residents are proven to utilize more follow-up services, ambulatory primary care visits, and less hospitalization in developed countries [5, 6, 9–11, 18, 19]. Though surveys found the first-visit to primary care might increase [20–24], we made a marginal contribution by using the indicator, the proportion of outpatient visits in community health centers, and directly revealing that the family doctor system changes the structure of outpatient care by attracting patients with hypertension and diabetes to community health centers and decreasing the health service utilization in secondary and tertiary hospitals. At the same time, the results revealed an increase in outpatient visits, exceeding the reduction in hospital admissions. This may indicate that in developing countries like China, after systematic optimization of service content within the family doctor system, the accessibility of healthcare services for patients has improved. This has led to the release of unmet medical needs, resulting in an increase in the utilization of related services in the short term [2, 32].

Under the family doctor system reform, the substitution effect between outpatient and inpatient care may exist [33–35]. A possible explanation is that chronic disease management services, such as regular examinations, follow-up services, and health education or counseling, prevent or delay further progress and diminish avoidable inpatient admissions. For example, controlling blood glucose can prevent renal failure in diabetic patients, thus avoiding the utilization of inpatient services due to renal failure [33]. From the societal perspective, the family doctor system may be beneficial to harnessing health expenditure growth and promoting population health.

Among patients with hypertension and diabetes, in our study, the family doctor system promotes the utilization of follow-up services and outpatient services but mitigates the increase in inpatient services. On the one hand, this result is consistent with former results from developed countries which verified more outpatient service utilization [5, 6, 10, 11]. On the other hand, unlike previous studies, our research findings do not support the hypothesis that the family doctor system reduces hospital service utilization [5]. We only observed that in the period of 2014–2017, while both the signed and non-signed groups experienced an increase in hospitalization rates, the family doctor system helped mitigate the growth rate of hospitalizations within the signed group. This may be attributed to the following reasons: Firstly, with the progress of universal health coverage in China, residents’ healthcare demands have been released and met, leading to an overall increase in various types of services including inpatient care. Secondly, the content of China’s family doctor system primarily focuses on strengthening chronic disease management and providing organized and continuous services, with implementation effects mainly concentrated on increasing follow-up and outpatient visits and optimizing the structure of outpatient care. Lastly, as the family doctor system has only recently been implemented in China, its impact on hospital service utilization requires more time, as the effect transmission exhibits a certain lag.

The family doctor system promotes a better structure of outpatient utilization among different institutions. Corresponding to our hypothesis, DID results show that after controlling other factors, compared with non-contracted patients, FD increases the health service utilization in community health centers, but decreases it in secondary and tertiary hospitals among contracted patients with hypertension and diabetes. In other words, FD effectively guides the reasonable diversion of patients to different medical institutions and attracts more patients to community health service centers. In China, the national policy requires “Patients firstly utilize primary care to distinguish patients with urgent or chronic diseases for referrals; establishing a two-way referral system linking the primary health centers with secondary or tertiary hospitals”, and family doctors and their services are identified as the cornerstone of primary health care and the entry point to the referral systems [17, 36]. At this stage, we proved that the family doctor system partly realizes the policy goal of referral systems and strengthens the function of community health centers by attracting more outpatient visits from secondary and tertiary hospitals [18–20]. However, we did not observe the change in emergency visits due to data limitations, while other studies found fewer emergency services were utilized among people with FDs than people without FDs [5, 7, 8, 12].

Under the backdrop of the family doctor system reform, the chronic disease management implemented in China has been shifting its focus from treatment to prevention and management. In China, before the implementation of the family doctor system, traditional management of chronic diseases was primarily carried out by secondary and tertiary hospitals, neglecting the role of community health centers in follow-up and daily management, which was characterized by fragmentation, lack of systematicity, and disorderliness. The family doctor system introduced a contract-based model for managing chronic diseases, which emphasized the role of community health centers and multidisciplinary team collaboration, to provide comprehensive services such as health assessments, regular follow-ups, health education, and medication management. As a result, by emphasizing prevention, early intervention, and continuous services, the family doctor system would facilitate rational management of chronic diseases, promote the development of primary care, and elevate patients’ health.

### Strengths and weaknesses of the study

This study fills the gap in China to quantitively evaluate the effect of the family doctor system for adequate observation time and adds to the evidence of the impact of FD on health service utilization with real-world data and appropriate methods. The study uses panel data to compare changes among contracted and non-contracted patients with hypertension and diabetes in health service utilization before and after the implementation of the family doctor system in real-world settings. The study combined two objective records with detailed information into the big sample size of 154,755 patients and then conducted the DID models and PSM method to rigorously evaluate this intervention with great validity.

Some limitations should be mentioned. Firstly, according to the comparison of the characteristics between contracted and non-contracted patients at baseline, there is a propensity whether a patient chooses to register with family doctors. Patients with hypertension and diabetes, who are female, elderly, with higher BMI and commodity of hypertension and diabetes, and enrolled in URRBMI intended to register with FD. It is consistent with previous Chinese research [37–39]. A possible reason is that the older the patients are, the worse their health condition, and the greater their motivation exists, and the more likely they register [32]. Our following study will also pay attention to the long-term impact of this selective behavior on health outcomes and disparity as a consensus mentioned in a local study [40]. Secondly, we cannot obtain more detailed data on emergency department utilization, medication usage, specific outpatient departments visited, diagnostic and treatment processes, and inpatient treatment, so we were unable to provide a comprehensive analysis of the overall impact of the family doctor system on healthcare utilization. The study can only draw conclusions regarding the optimization of outpatient utilization patterns but cannot infer policy implications regarding resource reallocation and the optimization of the referral system. Thirdly, due to a lack of relevant data, other variables that could reflect the severity of diseases among patients with hypertension and diabetes, were not collected. The severity of diseases was not included in the analysis and matching algorithms, and there may be other unobserved variables that could influence the outcome variables of this study. However, during the matching process, we included comorbidity as an indicator, primarily to capture the coexistence of diabetes and hypertension, attempting to partially reflect the complexity and severity of patients’ conditions. Nevertheless, it would be more accurate if we could conclude more related diseases such as heart disease, stroke, etc. Lastly, due to unavailable data in any other pre-treatment period earlier than 2014 as the baseline, the preliminary analysis cannot be conducted. To solve these two issues, the PSM method was conducted to balance the two groups as well as possible, which also showed robust results [41]. We will track the dynamic change of the family doctor system and evaluate its impact on health services in future studies.

### Implications for policy and practice

Three significant implications are instructive internationally as China and several developing countries consider strengthening gatekeepers in primary care reform. Firstly, drawing upon the experiences from pilot cities and the positive impact of the family doctor system, it is recommended to tailor and improve local regulations based on specific circumstances and implement the family doctor system nationwide. Secondly, considering the insufficient signing rate and selective signing, there is still much room for enhancing publicity across multiple channels so residents can improve awareness of the importance and necessity of contracting with family doctors [4, 42, 43]. Lastly, the key to enhancing the effectiveness of the family doctor system primarily lies in focusing on services related to standard, personalized chronic disease management. For the younger and healthier residents, providing health education, counseling, and individualized services is necessary to attract them to register with FDs [42]. For other higher-demand groups besides chronic disease patients, such as the poor and the disabled, identifying these populations and fully understanding their requirements are important to promote universal coverage and reduce health disparity [44, 45].

## Conclusions

In conclusion, this paper constructed Difference-in-Differences models based on the official health management records and medical insurance claim data of the patients with hypertension and diabetes to evaluate the family doctor system on health service utilization. The family doctor system increases follow-up visits and outpatient visits, and mitigates the increase inpatient admissions, and it also promotes a better structure of outpatient utilization with more outpatient visits in community health centers and fewer in secondary and tertiary hospitals. The examined family doctor system strengthens primary care, both by increasing follow-up visits and outpatient visits and promoting a rationalized structure of outpatient utilization in China.

## Data Availability

The data that support the findings of this study are available from the H city’s Health Information Center and the municipal Medical Insurance Bureau but restrictions apply to the availability of these data, which were used under license for the current study, and so are not publicly available.

## Abbreviations

PHC: Primary Health Care
FP: Family Physicians
GP: General Practitioner
FD: Family Doctors
DID: Differences-In-Differences
PSM: Propensity Score Matching
